# Unmet need for healthcare services among unemployed people – findings from a national survey in Finland

**DOI:** 10.1186/s12913-025-13765-8

**Published:** 2025-12-29

**Authors:** Hanna Rinne, Ari-Pekka Sihvonen, Visa Väisänen, Lars Leemann, Anna-Mari Aalto

**Affiliations:** 1 Rehabilitation Foundation, Pakarituvantie 5, Helsinki, 00410 Finland; 2 Finnish Institute for Health and Welfare, Welfare State Research Unit, Health and Social Service System Research Team, Mannerheimintie 166, Helsinki, 00330 Finland; 3 Faculty of Social Sciences and Business Studies, Department of Health and Social Management, University of Eastern Finland, Yliopistonranta 8 E, Kuopio, 70210 Finland; 4 Finnish Institute for Health and Welfare, Services Unit, Integrated Services Team, Mannerheimintie 166, Helsinki, 00330 Finland

**Keywords:** Unemployment, Unmet need, Healthcare services, Primary healthcare, Healthcare use, Employment status

## Abstract

**Background:**

Unemployed people have higher morbidity compared to employed people. Both the frequent use and the underutilization of healthcare services are common among unemployed people, potentially leading to unmet healthcare needs. We studied the differences in self-reported no need and unmet need for healthcare services between unemployed and employed persons and the health-related factors associated with these differences.

**Methods:**

We used the Healthy Finland Survey of 2022–2023, which included employed and unemployed respondents of working age (20–64, *N* = 9841). The outcomes were (1) no need for healthcare services and (2) unmet need for healthcare services in the previous 12 months. We used logistic regression, adjusting for sociodemographic and health-related factors, including limiting long-term illness, psychological distress, limited functional ability, very weak social inclusion, poor work ability, smoking, and excessive alcohol consumption.

**Results:**

Among unemployed people, 23% reported no need for a doctor or nurse, whereas 41% reported unmet needs. Compared with employed people, unemployed people were more likely to perceive no need for doctor or nurse services (OR 1.36, 95% CI 1.02–1.82). When health-related factors, especially long-term illness and work ability, were controlled for, the difference increased. In the full model, the OR was 1.87 (95% CI 1.35–2.58). Unemployed people were more likely than employed people to have unmet need for doctor or nurse (OR 2.31, 95% CI 1.78–3.00). For most of the health-related factors, especially work ability, controlling decreased the difference, whereas controlling for smoking and alcohol had little effect. In the full model the OR was 1.33 (95% CI 0.98–1.81).

**Conclusions:**

Unmet healthcare need among unemployed people was largely attributable to health-related factors, especially poor work ability. In some cases, unemployed people may not recognize their need for healthcare, highlighting the importance of low-threshold services and proactive outreach to ensure equitable access. Healthcare systems should ensure effective and timely use of services to prevent diagnostic delays or untreated illnesses and support more appropriate use of sickness benefits and rehabilitation services.

## Introduction

Countries worldwide are undergoing major demographic shifts due to population aging [1] and declining birth rates [2]. These trends, along with rising dependency ratios, pose challenges to public finances, labour markets, and the sustainability of welfare systems [3, 4]. Austerity policies [5, 6] and the growing burden of chronic diseases [7] further strain access to healthcare services and increase care needs [8]. In ageing societies, higher labour force participation and longer working lives are essential [9]. To maintain population health and the sustainability of welfare systems, it is crucial to meet healthcare needs while supporting work ability—especially among vulnerable groups such as unemployed people. Therefore, understanding the factors behind healthcare needs and unmet needs among unemployed people is vital for promoting both employment and well-being.

Several studies have shown that unemployed people tend to have poorer physical and mental health than those who are employed. While those who become unemployed are often already in worse health, unemployment itself has been found to further deteriorate health [10–15] — particularly mental health [15, 16]. A prolonged duration of unemployment exacerbates the detrimental effects on health [14, 16, 17]. In addition, unemployment is associated with reduced psychological and physical functioning [10] and weak social inclusion [18], which may impair work ability defined as a balance between personal resources and job demands [19] and contribute to social exclusion. It can also negatively influence health behaviours [16, 20].

The health and work ability of unemployed people, and thus their employment, can be promoted through appropriate healthcare services. However, seeking healthcare services is not merely based on impaired health but also requires the ability to perceive and recognize needs, demand and reach appropriate services, and to engage with healthcare services once accessed [21]. The availability, accessibility, and acceptability of services also play key roles in healthcare services use. Both individual and systemic factors affect whether people have access to and actually use healthcare services.

Unemployment is associated with increased use of mental health services [22, 23], but its impact on primary and hospital care use is unclear [23]. The overall use of healthcare services may slightly decline after job loss [16]. Unemployment is also associated with not seeking healthcare services among those with fair or poor health [24] and being less likely to receive adequate treatment among those with poor mental health [25, 26]. Unemployed people with depressive symptoms may avoid seeking healthcare [27], leading to undiagnosed conditions, particularly psychiatric disorders [28]. The structure of the healthcare system matters: universal systems reduce negative effects, but employed people with broader healthcare coverage still tend to have better access [23]. Job loss increases the risk of unmet healthcare needs; however, these effects are less pronounced in countries with low copayments and generous unemployment benefits [29]. Finally, unemployed people face more access barriers [30], for example, high out of pocket payments and a poor financial situation are common reasons for reporting high unmet needs [31, 32].

### Finnish context

In the tax-funded Finnish healthcare system, social and healthcare services are arranged by 21 wellbeing county services and the city of Helsinki, mostly provided in public healthcare centers and public hospitals [33]. The role of nurses is emphasized, and they, in principle, act as gatekeepers to physicians, which in turn act as gatekeepers to specialized healthcare services. The system is supplemented by a reimbursement scheme for private services, in addition to a unique occupational healthcare system, which provides primary healthcare for employed people with better access and without out-of-pocket payments [34].

Access to healthcare services varies significantly on the basis of employment status. While employed people benefit from occupational healthcare with fast access free of costs, unemployed people rely primarily on public healthcare services, which often involve longer waiting times and substantial out-of-pocket costs. Private healthcare is also used more frequently by employed people [34–38]. Previous research has shown that unemployed people use either fewer [35, 36] or similar amounts [38] of healthcare services than employed people do. However, after job loss, service use tends to decline, as occupational healthcare is not replaced by other services [39]. There is also variation in the use of healthcare services among unemployed people [23]. Factors such as gender, perceived health, economic situation, residential area, marital status, and duration of unemployment influence access and utilization [36]. In particular, among long-term unemployed the use of healthcare services is polarised— some use services extensively, whereas others do not at all [40–42]. Notably, a quarter of unemployed people used no primary or social care services during a year, and 10% account for 60% of total care costs [43]. Unemployed persons are also overrepresented among frequent users of public outpatient healthcare services [44]. According to the OECD [45], unemployment among younger individuals in Finland is often associated to low education, limited work experience and mental health problems, while older unemployed people may have outdated skills, health issues, and age-related barriers in the labour market.

### Unmet need for healthcare services

Healthcare service use does not always indicate whether people’s care needs are actually met. Self-perceived unmet need is considered a key indicator of equal access to healthcare services [46]. However, there is no agreed-upon definition of unmet healthcare need [47]. Unmet need can be divided into five types: (1) unperceived unmet need, (2) subjective, chosen unmet need, (3) subjective, non-chosen unmet need, (4) subjective, clinician-validated unmet need, and (5) subjective unmet expectations [48]. Unmet need may involve not receiving any care or receiving inadequate care, often due to limited availability, affordability, or quality of services [47, 49]. Importantly, not all unmet needs are reported in surveys, as people who are unaware of their need or who choose not to seek care may not report it. Similarly, healthy individuals who do not need care are not considered to have unmet needs. Therefore, it is crucial to also study those who do not seek healthcare at all [47].

Compared with employed people, unemployed are found to have higher risk for unmet need in Finland [50–52], in Sweden [27, 31] and in other countries [53–56]. Few studies examining the differences in unmet need between unemployed and employed people have considered a wide range of health-related factors. Some studies have included only self-rated health [50, 51], whereas some have added also other factors: morbidity [53, 56], selected long-term illnesses and quality of life [52], long-term illness, depressive feelings, exhaustion, social networks [27], and trust [31]. A Luxembourgian study investigated the components of unmet need and took several health-related factors into account such as body mass index, smoking, alcohol consumption, chronic diseases and limitations in activities due to health problems [32]. In general, adverse health-related factors are associated with more common unmet need [57]. In Finland, a higher risk of unmet need has also been associated with younger age, female gender, non-Finnish language background, being outside the labour force, need for income support and living in urban areas [51].

Effective utilization of healthcare services is essential, not only for timely treatment but also for securing sickness benefits. Unemployed people may miss these benefits because of undiagnosed conditions and receive less support during sickness allowance periods, resulting in unmet rehabilitation needs. Both the frequent use and underutilization of healthcare services are common among unemployed people. However, there is not much research on unemployed people who do not experience the need healthcare services at all. Among the studies on the unmet need for healthcare services, very few examined the effects of different health-related factors.

### Aims

We studied the differences in self-reported no need and unmet need for healthcare services between unemployed and employed people and the health-related factors associated with these differences. The detailed research questions were:

- Is there a difference in the prevalence of self-reported no need and unmet need for healthcare services among unemployed and employed people?
- How are different health-related factors related to the differences between unemployed and employed people in self-reported no need and unmet need for healthcare services?
- Are there differences in self-reported no need and unmet need for healthcare services by the duration of unemployment or by age group?

## Methods

### Data and study population

The experiences of unemployed people in using healthcare services and the factors associated with them were studied using the Healthy Finland Survey carried out by THL in 2022–2023. The Healthy Finland is a regularly conducted large cross-sectional national survey providing reliable, nationally representative, and up-to-date information on the health, well-being, and service use of adults living in Finland [58, 59]. The original English versions of the questionnaires can be found on the THL website [60]. The sample was randomly selected and stratified by wellbeing service counties (21 counties and Helsinki capital city as an independent region). From each county, 2 800 persons (2 000 persons aged 20–74 years and 800 aged 75 or older) were drawn from the population registry maintained by the Digital and Population Data Service Agency (DVV). The questionnaire was tailored according to age group (20–54 years / 55–74 years / 75 years or older). The survey explored the well-being and health of adults aged 20 and over living in Finland and their experiences with social and health services. The response rate was 46%.

The present study was restricted to working aged (20–65 years) respondents (N = 12,976). The study population consisted of 9,841 people who were employed, 93% (n = 9,138) of whom were employed part- or full-time and 7% (n = 703) were unemployed or laid off. Unemployment was defined based on respondents’ self-reported labour market status. Participants were asked to select the option that best described their current situation, including categories such as ‘unemployed or laid off’, ‘retired’, ‘receiving a disability pension or rehabilitation benefit’, or ‘student’, allowing for a distinction between those unemployed and those outside the labour force. The proportion of unemployed individuals in our sample (7%) was in line with the official unemployment rates in Finland during the data collection period [61].

### Outcomes

We had two outcomes: no need for healthcare services and an unmet need for healthcare services.

The variables were based on the same question: “Do you feel you have received enough of the following healthcare services in the previous 12 months? Please note services provided by the municipality, occupational services and private service providers.” The items in the battery of questions included “doctor’s appointment services” and “nurse’s or public health nurse’s appointment services”. The response options were as follows: (1) I have not needed these services; (2) I would have needed these services, but could not access them; (3) I have used the services, but they were not adequate; and (4) I have used the services and they were adequate.

The outcome for not needing healthcare services included all respondents and was dichotomized as follows: 1) I have not needed these services, 0) other. The outcome unmet need included only those who have needed services: 1) I would have needed these services but could not access them or I have used the services, but they were not adequate, 0) I have used the services and they were adequate.

### Health-related factors

The examined health-related factors included limiting long-term illness, psychological distress, limited functional ability, very weak social inclusion, poor work ability, daily smoking and excessive alcohol consumption (Table 1).

**Table 1:** Health-related factors examined

Limiting long-term illness	Global activity limitation indicator, GALI. Limitation in activities because of long-standing health problems. [62, 63] Categories: 0) no, 1) yes
Psychological distress	The Mental Health Inventory, MHI-5, 5 questions about anxiety, depression and positive mood during the past 4 weeks. MHI-5 score ≤ 52. [64]. Categories: 0) no (MHI-5 score > 52), 1) yes (MHI-5 score ≤ 52)
Limited functional capacity	Unable to perform one or more of the following 8 items: walk approximately 500 m without stopping to rest, see ordinary newspaper print, hear what is said in a conversation between several people, memory is good, absorbing new information and learning things, can usually concentrate on things, walk up one flight of stairs, run a short distance (approximately 100 m). [65] Categories: 0) no, 1) yes
Very weak social inclusion	Experiences of Social Inclusion Scale (ESIS), 10 statements based on a comprehensive framework on social inclusion. ESIS scores below 50 refer to a very weak experience of social inclusion. [66] Categories: 0) no (ESIS score 50–100), 1) yes (ESIS score < 50).
Poor work ability	Self-reported ability to work is impaired/reduced/limited “Regardless of whether you are employed or not, please estimate your current work capacity. Are you: completely fit for work; partially unable to work; completely unable to work?” [67] Categories: 0) no (“completely fit for work”), 1) yes (“partially or completely unable to work”)
Excessive alcohol consumption	The Alcohol Use Disorders Identification Test AUDIT-C. [68] Categories: 0) no, 1) yes (≥ 6 for men and ≥ 5 for women)
Daily smoking	Daily tobacco or nicotine product user. Categories: 0) no, 1) yes

### Sociodemographic factors

The controlled sociodemographic factors were sex, age, language, education, living alone and degree of urbanization. The variable categories are detailed in Table 3. In the models, age was categorized into 5-year age groups. Information about education and living alone was asked about in the survey. All other information was previously merged from the registers of Statistics Finland. Additionally, unemployed individuals were categorized on the basis of the duration of their unemployment: less than 12 months, 12 months or more, and cases with missing duration data.

### Statistical methods

Descriptive analysis was performed using frequencies, percentages, and cross-tabulations. Univariate and multivariate logistic regression approaches were used to estimate the effects of each variable while controlling for others. Missing data was handled by pairwise deletion. Inverse probability weights were used as a statistical method in the analyses to adjust for differences in selection probability and to reduce the bias due to non-participation. Non-participation was more common among men, participants of younger age, and those with lower levels of education [59]. Construction of the weights in The Healthy Finland Survey is described in more detail elsewhere [59, 69]. The outcome variables were no need or unmet need for doctor and nurse services. First, we fitted the models including only employment status. Second, we adjusted for sociodemographic factors and then each health-related factor at a time. Finally, we included all sociodemographic and health-related factors at the same time. There was no multicollinearity between the variables. Models were fitted for the entire study population, comparing all unemployed individuals with those who were employed. We also examined unemployed people on the basis of the duration of their unemployment. In addition, comparisons between unemployed and employed people were conducted by age group (25–34 and 50–64 years). Statistical analysis was performed with Stata version 15.1.

## Results

Differences in having no need for doctor or nurse services were not found between unemployed and employed people (Table 2). 23% of unemployed people reported no need for doctor or nurse. However, unemployed people had higher levels of unmet need for doctor or nurse services than employed people. 41% of unemployed people had an unmet need for healthcare services, whereas the corresponding figure for employed people was 23%.

**Table 2:** Need for Doctor or nurse services (%) among unemployed and employed people

	Unemployed		Employed	
	%	95% CI	%	95% CI
Need for doctor*				
I have not needed these services	29	(24–34)	25	(23–26)
I would have needed these services, but could not access them	11	(8–14)	5	(5–6)
I have used the services, but they were not adequate	18	(14–22)	11	(10–12)
I have used the services and they were adequate	42	(37–48)	59	(58–61)
Total	100		100	
Need for nurse*				
I have not needed these services	38	(32–43)	36	(35–37)
I would have needed these services, but could not access them	7	(5–10)	2	(2–3)
I have used the services, but they were not adequate	10	(8–14)	7	(7–8)
I have used the services and they were adequate	45	(40–50)	54	(53–56)
Total	100		100	
**Outcome variables**				
No need for doctor and nurse	23	(19–28)	19	(18–20)
Unmet need for doctor or nurse	41	(36–47)	23	(22–24)
N	703		9138	

*Answers for the question: ‘Do you feel you have received enough of the following healthcare services in the previous 12 months’

Compared with employed people, unemployed people were more likely to be male, at the low or high end of the age distribution, speak a foreign language, have a low education, and live alone (Table 3). There were no differences in the degree of urbanisation of the residential area. All health-related outcomes indicated worse health and functioning among unemployed people than among employed people.

Men, 20–34-year-olds, and Swedish speakers had more often no need for doctor and nurse services than their counterparts. With respect to health-related factors, no need for doctor and nurse services was less common among those with limiting long-term illnesses, psychological distress, limited functional capacity, and poor work ability, whereas there was no difference in terms of weak social inclusion, daily smoking nor excessive alcohol consumption.

Women and non-Finnish speakers had more often unmet need for doctor and nurse services than their counterparts. In terms of health-related factors, the unmet need for doctor and nurse services was more common among those with limiting long-term illness, psychological distress, limited functional capacity, poor work ability, weak social inclusion, and daily smoking. There was no difference regarding excessive alcohol consumption.

**Table 3:** Sociodemographic and health-related factors, and healthcare needs (%) among unemployed and employed people

	Unemployed % (95% CI)	Employed % (95% CI)	Prevalence of no need % (95% CI)	Prevalence of unmet need % (95% CI)
**Sex**				
Male	61 (56–66)	51 (50–53)	24 (23–26)	22 (20–24)
Female	39 (34–44)	49 (50–53)	14 (13–15)	27 (25–28)
**Age**				
20–34	34 (29–34)	28 (27–29)	24 (22–27)	26 (23–29)
35–49	29 (25–34)	38 (37–40)	20 (18–22)	25 (23–27)
50–64	37 (33–42)	34 (33–35)	15 (14–16)	22 (21–24)
**Language**				
Finnish	80 (73–84)	87 (85–88)	19 (18–20)	23 (22–25)
Swedish	3 (2–6)	4 (4–5)	29 (24–35)	23 (18–29)
Other	17 (13–23)	9 (8–10)	20 (15–25)	33 (27–40)
**Education**				
Low	53 (48–58)	40 (39–42)	21 (19–23)	26 (24–28)
Middle	27 (23–32)	32 (31–33)	19 (17–20)	23 (21–25)
High	19 (16–24)	28 (27–29)	18 (16–20)	23 (21–25)
**Living alone**				
Yes	42 (37–48)	23 (22–24)	22 (19–25)	26 (23–29)
No	58 (52–63)	77 (76–78)	19 (18–21)	24 (23–26)
**Degree of urbanisation**				
Urban	79 (75–82)	76 (75–77)	20 (18–21)	24 (23–26)
Densely populated	11 (8–14)	14 (13–15)	18 (15–20)	24 (21–27)
Rural	11 (8–14)	10 (9–11)	21 (18–24)	25 (22–28)
**Limiting long-term illness**				
Yes	44 (39–49)	25 (24–26)	8 (7–9)	37 (35–40)
No	56 (51–61)	75 (74–76)	23 (22–25)	19 (17–20)
**Psychological distress**				
Yes	37 (32–42)	15 (14–16)	15 (13–18)	40 (36–44)
No	63 (58–68)	85 (84–86)	20 (19–22)	21 (20–22)
**Limited functional capacity**				
Yes	32 (27–36)	13 (12–14)	12 (10–14)	38 (35–42)
No	68 (64–73)	87 (86–88)	21 (20–22)	22 (20–23)
**Very weak social inclusion**				
Yes	29 (24–34)	7 (6–7)	19 (15–23)	43 (38–49)
No	71 (66–76)	93 (93–94)	19 (18–21)	22 (21–24)
**Poor work ability**				
Yes	49 (43–54)	10 (9–11)	9 (7–12)	44 (41–48)
No	51 (46–57)	90 (89–91)	21 (20–22)	21 (20–22)
**Excessive alcohol consumption**				
Yes	30 (26–34)	25 (24–26)	20 (18–22)	22 (20–24)
No	70 (66–74)	75 (74–76)	19 (18–21)	25 (23–26)
**Daily smoking**				
Yes	25 (21–29)	14 (13–15)	22 (19–25)	29 (26–33)
No	75 (71–79)	86 (85–87)	19 (18–20)	23 (22–25)
**Total %**	100	100	*19 (18–20)*	*24 (23–26)*
**N**	*703*	*9138*		

Compared with employed people, unemployed people reported slightly more often (OR: 1.36, CI: 1.02–1.82) no need for doctor or nurse services (Table 4, univariate model). Adjusting for sociodemographic factors reduced the odds ratio to 1.25 (CI: 0.93–1.67), rendering the association statistically nonsignificant (Model 1). Adjusting for sociodemographic factors and limiting long-term illness (OR: 1.57, CI: 1.16–2.12) or poor work ability (OR: 1.82, CI: 1.32–2.50) increased the difference between employed and unemployed people considerably, whereas adjusting for psychological distress or limited functional capacity increased the difference only slightly (OR: 1.36, CI: 1.02–1.84 and OR: 1.37, CI: 1.02–1.84, respectively). Adjusting for weak social inclusion, daily smoking or excessive alcohol consumption did not have an effect. (Models 2a–2 g.) Adjusting for all sociodemographic and health-related factors increased the difference between unemployed and employed people to an odds ratio of 1.87 (CI: 1.35–2.58) (Model 3).

There was no difference in the strength of the association by duration of unemployment. Overall, the association between unemployed and employed people with no need for doctor and nurse services were similar but slightly stronger among 25─34-year-olds than among all unemployed people and weak or non-existing among the 50─64-year-olds.

Unemployed people reported significantly more often (OR: 2.31, CI: 1.78–3.00) unmet need for doctor or nurse services than employed people (Table 5, univariate model). Adjusting for sociodemographic factors did not change the odds ratio (Model 1). Adjusting for sociodemographic factors and poor work ability (OR: 1.55, CI: 1.16–2.08) considerably decreased the difference between employed and unemployed people, whereas adjusting for limiting long-term illness, psychological distress, limited functional capacity or poor social inclusion moderately decreased the difference (ORs ranging between 1.88 and 2.05) (models 2a–2 g). Adjusting for daily smoking or excessive alcohol consumption had little effect. Adjusting for sociodemographic and all health-related factors explained a large proportion of the differences between unemployed and employed people, resulting in an odds ratio of 1.33 (CI: 0.98–1.81) (Model 3).

Overall, those who had been unemployed for at least 12 months had higher risk of unmet need for doctor or nurse services than those who had been unemployed for less than 12 months. The association between unemployed and employed people in unmet need for doctor and nurse services was weaker among the 25─34-year-olds than among the 50─64-year-olds or among all unemployed people (but not statistically significant based on the overlapping confidence intervals), and non-existent when adjusting for sociodemographic and all health and risk-behaviour related factors (Model 3).

**Table 4:** No need for Doctor and nurse services among unemployed and employed people (OR)

	Univariate models	Model 1 Socio-demographic factors	Model 2a Limiting long-term illness	Model 2b Psychological distress	Model 2c Limited functional capacity	Model 2d Poor work ability	Model 2e Weak social inclusion	Model 2f Daily smoking	Model 2g Excessive alcohol consumption	Model 3 Full multivariate model
	OR (95% CI)	OR (95% CI)	OR (95% CI)	OR (95% CI)	OR (95% CI)	OR (95% CI)	OR (95% CI)	OR (95% CI)	OR (95% CI)	OR (95% CI)
**25-64-year- olds**										
Employed	1.00	1.00	1.00	1.00	1.00	1.00	1.00	1.00	1.00	1.00
Unemployed	1.36 (1.02–1.82)	1.25 (0.93–1.67)	1.57 (1.16–2.12)	1.36 (1.02–1.83)	1.37 (1.02–1.84)	1.82 (1.32–2.50)	1.32 (0.98–1.78)	1.26 (0.94–1.68)	1.25 (0.94–1.68)	1.87 (1.35–2.58)
**25-64-year- olds**										
Employed	1.00	1.00	1.00	1.00	1.00	1.00	1.00	1.00	1.00	1.00
Unemployed < 12 Mo	1.57 (1.00–2.48)	1.39 (0.87–2.21)	1.68 (1.04–2.71)	1.47 (0.92–2.35)	1.45 (0.91–2.31)	1.72 (1.05–2.82)	1.43 (0.90–2.28)	1.40 (0.88–2.23)	1.39 (0.87–2.22)	1.83 (1.12–3.01)
Unemployed ≥ 12 M	1.34 (0.86–2.10)	1.30 (0.83–2.04)	1.65 (1.06–2.58)	1.42 (0.90–2.23)	1.44 (0.92–2.26)	2.01 (1.25–3.22)	1.38 (0.88–2.18)	1.31 (0.83–2.06)	1.31 (0.83–2.05)	2.01 (1.26–3.19)
Unemployed dur. missing	1.32 (0.82–2.13)	1.21 (0.76–1.91)	1.55 (0.96–2.50)	1.37 (0.87–2.17)	1.40 (0.88–2.21)	2.06 (1.23–3.45)	1.30 (0.82–2.07)	1.22 (0.77–1.93)	1.21 (0.77–1.92)	2.04 (1.23–3.38)
**25-34-year- olds**										
Employed	1.00	1.00	1.00	1.00	1.00	1.00	1.00	1.00	1.00	1.00
Unemployed	1.60 (0.95–2.70)	1.55 (0.90–2.67)	1.98 (1.12–3.48)	1.70 (0.98–2.94)	1.65 (0.96–2.83)	1.91 (1.07–3.41)	1.67 (0.95–2.93)	1.59 (0.93–2.74)	1.56 (0.91–2.68)	2.10 (1.15–3.85)
**50-64-year- olds**										
Employed	1.00	1.00	1.00	1.00	1.00	1.00	1.00	1.00	1.00	1.00
Unemployed	1.00 (0.69–1.44)	0.92 (0.63–1.36)	1.22 (0.82–1.81)	1.01 (0.68–1.49)	1.07 (0.72–1.59)	1.50 (0.99–2.28)	0.98 (0.66–1.44)	0.92 (0.62–1.36)	0.92 (0.63–1.36)	1.51 (1.00–2.27)

N=8,238 for 25─64-year-olds, N = 1,655 for 25─34-year-olds, and N = 3,790 for 50─64-year-olds

Univariate models included only employment status

Models 1 and 2a─2g were adjusted for sex, age, language, education, living alone, degree of urbanization and each health-related factor at a time

Model 3 was adjusted for sex, age, language, education, living alone, and degree of urbanization, as well as for the health-related factors of models 2a─2g

**Table 5:** Unmet need for Doctor or nurse services among unemployed and employed people (OR)

	Univariate models	Model 1 Socio-demographic factors	Model 2a Limiting long-term illness	Model 2b Psychological distress	Model 2c Limited functional capacity	Model 2d Poor work ability	Model 2e Weak social inclusion	Model 2f Daily smoking	Model 2g Excessive alcohol consumption	Model 3 Full multivariate model
	OR (95% CI)	OR (95% CI)	OR (95% CI)	OR (95% CI)	OR (95% CI)	OR (95% CI)	OR (95% CI)	OR (95% CI)	OR (95% CI)	OR (95% CI)
**25-64-year- olds**										
Employed	1.00	1.00	1.00	1.00	1.00	1.00	1.00	1.00	1.00	1.00
Unemployed	2.31 (1.78–3.00)	2.31 (1.75–3.05)	1.88 (1.42–2.49)	1.99 (1.49–2.66)	2.05 (1.54–2.73)	1.55 (1.16–2.08)	1.92 (1.43–2.57)	2.25 (1.70–2.99)	2.32 (1.76–3.07)	1.33 (0.98–1.81)
Employed	1.00	1.00	1.00	1.00	1.00	1.00	1.00	1.00	1.00	1.00
Unemployed < 12 Mo	1.83 (1.17–2.87)	1.76 (1.09–2.82)	1.46 (0.90–2.36)	1.57 (0.94–2.62)	1.67 (1.04–2.70)	1.38 (0.85–2.26)	1.54 (0.94–2.53)	1.72 (1.07–2.78)	1.76 (1.10–2.84)	1.20 (0.72–2.00)
Unemployed ≥ 12 M	2.89 (1.96–4.25)	3.12 (2.12–4.60)	2.53 (1.71–3.77)	2.73 (1.85–4.04)	2.72 (1.82–4.08)	1.96 (1.31–2.93)	2.56 (1.70–3.85)	3.04 (2.05–4.51)	3.14 (2.14–4.62)	1.74 (1.14–2.65)
Unemployed dur missing	2.48 (1.60–3.85)	2.42 (1.51–3.89)	1.97 (1,25–3.11)	1.99 (1.23–3.23)	2.06 (1.26–3.36)	1.52 (0.96–2.41)	1.96 (1.20–3.20)	2.36 (1.46–3.81)	2.45 (1.53–3.92)	1.29 (0.80–2.08)
**25-34-year- olds**										
Employed	1.00	1.00	1.00	1.00	1.00	1.00	1.00	1.00	1.00	1.00
Unemployed	1.79 (1.07–3.01)	1.73 (1.03–2.94)	1.44 (0.84–2.45)	1.51 (0.87–2.62)	1.65 (0.97–2.80)	1.23 (0.71–2.12)	1.57 (0.91–2.70)	1.69 (0.99–2.89)	1.75 (1.04–2.94)	1.06 (0.59–1.89)
**50-64-year- olds**										
Employed	1.00	1.00	1.00	1.00	1.00	1.00	1.00	1.00	1.00	1.00
Unemployed	2.51 (1.81–3.48)	2.44 (1.72–3.45)	1.93 (1.35–2.78)	2.17 (1.53–3.08)	2.01 (1.41–2.86)	1.64 (1.12–2.42)	1.99 (1.39–2.83)	2.42 (1.70–3.44)	2.45 (1.73–3.47)	1.44 (0.97–2.13)

N = 6,762 for 25─64-year-olds, N = 1,260 for 25─34-year-olds, and N = 3,234 for 50─64-year-olds

Univariate models included only employment status

Models 1 and 2a-2g were adjusted for sex, age, language, education, living alone, degree of urbanization and each health-related factor at a time

Model 3 was adjusted for sex, age, language, education, living alone, and degree of urbanization, as well as for the health-related factors of models 2a─2g

Next, the associations between health-related factors and having no or unmet need for doctor or nurse services were explored. According to the univariate models, most health-related factors were associated with higher odds of experiencing no need or unmet needs for doctor or nurse services (Table 6). In the full multivariate models, limiting long-term illness was strongly associated with both lower odds of having no need and increased odds of unmet need (OR: 0.34, CI: 0.28–0.42 and OR: 1.99, CI: 1.68–2.36, respectively). Similarly, work ability emerged as an important factor for both no need and unmet needs (OR: 0.56, CI: 0.39–0.79 and OR: 1.77, CI: 1.42–2.21, respectively). Psychological distress was associated with increased unmet needs (OR: 1.57, CI: 1.25–1.97).

**Table 6:** Need for Doctor or nurse by employment status and health-related factors (OR)

	No need (doctor or nurse)		Unmet need (doctor or nurse)	
	Univariate models	Multivariate model	Univariate models	Multivariate model
	OR (95% CI)	OR (95% CI)	OR (95% CI)	OR (95% CI)
**Employment status**				
Employed	1.00	1.00	1.00	1.00
Unemployed	1.25 (0.93–1.67)	1.87 (1.35–2.58)	2.31 (1.75–3.05)	1.33 (0.98–1.81)
**Limiting long-term illness**				
No	1.00	1.00	1.00	1.00
Yes	0.28 (0.22–0.34)	0.34 (0.28–0.42)	2.61 (2.23–3.03)	1.99 (1.68–2.36)
**Psychological distress**				
No				
Yes	0.60 (0.48–0.77)	0.77 (0.59–1.02)	2.25 (1.85–2.75)	1.57 (1.25–1.97)
**Limited functional capacity**				
No	1.00	1.00	1.00	1.00
Yes	0.49 (0.38–0.62)	0.75 (0.57–0.99)	2.07 (1.71–2.,50)	1.22 (0.99–1.51)
**Poor work ability**				
No	1.00	1.00	1.00	1.00
Yes	0.32 (0.23–0.43)	0.56 (0.39–0.79)	2.97 (2.44–3.59)	1.77 (1.42–2.21)
**Very weak social inclusion**				
No	1.00	1.00	1.00	1.00
Yes	0.77 (0.57–1.04)	1.24 (0.87–1.77)	2.27 (1.71–3.03)	1.34 (0.98–1.83)
**Daily smoking**				
No	1.00	1.00	1.00	1.00
Yes	0.91 (0.73–1.13)	1.00 (0.79–1.26)	1.23 (0.99–1.55)	1.18 (0.93–1.51)
**Excessive alcohol consumption**				
No	1.00	1.00	1.00	1.00
Yes	0.93 (0.74–1.15)	0.99 (0.82–1.19)	0.89 (0.75–1.06)	0.78 (0.65–0.93)

No need *N* = 8,238, unmet need *N* = 6,762. All the models, including the univariate models, were adjusted for sex, age, language, education, living alone, and degree of urbanization

## Discussion

### Main results

This study analysed the differences in self-reported no need and unmet need for healthcare services between unemployed and employed people and examined the health-related factors associated with these differences. The outcomes of no need and unmet need were examined as separate processes with potentially different mechanisms. Unemployed people could report no need for services due to poor previous experiences, perceiving that the healthcare system is not able to assist with their problems, or simply having no needs. On the contrary, unmet needs more clearly indicate a mismatch between perceived care needs and received services.

The differences between unemployed and employed people in terms of perceived no need for a doctor or nurse services were minor. However, when accounting for health-related factors, particularly long-term illness and work ability, reporting no need for healthcare services was more common among unemployed people. The difference was stronger among those aged 20–34. The duration of unemployment was not associated with reporting no need for healthcare services.

Unemployed people were more likely than employed people to have unmet needs for doctor or nurse services. Controlling for most health-related factors, especially work ability, reduced this difference, whereas controlling for smoking and alcohol had a minimal effect. Unmet need was more pronounced among those with longer unemployment and among 50–64-year-olds. When the association of health-related factors on no or unmet needs were analysed, limiting long-term illness and work ability emerged as the strongest predictors of both having no need and having unmet needs for healthcare services.

### Interpretation

Unemployed people often report no need for healthcare services, which may reflect a range of underlying factors. It is important to distinguish between a genuine lack of need and passive no use despite an objective need for healthcare. Higher morbidity, especially chronic disease, among unemployed people may lead to persistent symptoms becoming normalized, reducing the perceived urgency of seeking healthcare. Some long-term illnesses may be under control and in addition to ongoing care, unemployed people might not perceive additional healthcare needs. However, this explanation remains somewhat speculative, and more evidence is needed to support this interpretation. Barriers such as limited knowledge of the healthcare system, doubts about its usefulness, financial difficulties, and long travel times may cause unemployed people to report having no need for healthcare services [21, 70]. In contrast to employer-sponsored occupational healthcare services, which often meet the more straightforward needs of employed people, the complex and multifaceted needs of unemployed people may require integrated and multidisciplinary care [71].

Stigma and shame can deter unemployed people from seeking healthcare, especially unemployed people with mental health problems [72]. As in previous studies [25–27], this study also suggested that mental health problems may underlie the high prevalence of perceived no need for healthcare services. Psychological distress did not explain the differences between unemployed and employed people overall but was related to differences within the youngest age group. Unemployment may also narrow social networks that typically support access to healthcare services [23]. However, when weak social inclusion was considered, it did not explain the differences in perceived no need for healthcare services between unemployed and employed people. This suggests the relevance of other contextual factors, such as the role of employment itself. Employed people often require quicker access to healthcare to maintain their work ability. They may be encouraged by supervisors or colleagues and have specific work-related healthcare needs, such as medical certificates for sickness absences or occupational safety related health problems. Unemployed people, with fewer such requirements, may delay seeking healthcare even when it would be needed.

Although the difference in unmet need for healthcare services between unemployed and employed people was explained by health-related factors, this does not imply that access to healthcare services is sufficient for those with greater health needs. Rather, it may reflect that unemployed people with greater healthcare need are not sufficiently reached by existing services. These results have several potential interpretations. First, the Finnish healthcare system has been criticized for inequity in access to healthcare because most employed people have better access to primary healthcare services through occupational healthcare whereas unemployed people rely on public services with long waiting times [34]. Therefore, the results may reflect actual difficulties in accessing healthcare services. Second, healthcare needs among unemployed people are often more complex, which might make it difficult for both individuals and healthcare professionals to recognize and respond [73]. This may result in healthcare being perceived as inadequate and insufficient, which is especially relevant if care pathways are difficult to navigate. While in Finland, public health centres in principle offer integrated care services and utilize multidisciplinary teams [74], in other countries with more fragmented social and healthcare systems, meeting the complex care needs of unemployed people might be difficult. Third, those employed may be more motivated to seek urgent healthcare to maintain the ability to work. Social networks have been shown to affect healthcare utilization by providing relevant information and setting social norms [75]. Coworkers and other social contacts may support timely care seeking among those employed, whereas unemployed people may have less supportive social networks.

Importantly, unemployed people are a heterogeneous group, especially in terms of their care needs. Previous research has indicated that unemployed people have multiple profiles of healthcare use, with some having less morbidity and no service use, whereas others are frequent attenders in multiple service types [43, 76]. Our results suggest a similar trend, as unemployed people do not differ much from employed people in terms of not needing healthcare services. The difference in reporting no need was more pronounced when considering unemployed people’s poorer health. On the other hand, among those who used healthcare services, unmet need was more common for unemployed people, but the association was at least partly mediated by worse health. The healthcare needs of unemployed people also differed by age group. The difference in reporting no need for healthcare services between unemployed and employed people was slightly greater among younger people than in the oldest age group. Moreover, older unemployed people experienced higher unmet healthcare needs driven mainly by limited functional capacity and work ability. Similarly, a longer duration of unemployment was associated with unmet needs. These results highlight the need for various tailored and person-centered measures and interventions to reduce unmet healthcare needs among unemployed people [21, 71].

Reduced work ability was a key determinant of unmet healthcare needs among unemployed people. In healthcare services, it is essential to assess not only medical conditions but also individuals’ work ability [77]. However, evaluating work ability in unemployed individuals is more challenging because of to the absence of concrete job tasks. Moreover, public primary healthcare may lack sufficient expertise in work ability assessments [78]. Targeted health examinations offer one approach for assessing the work and functional capacity of unemployed individuals. However, the implementation of these examinations is inconsistent, and significant gaps remain in evaluating both work ability and the need for supportive measures [79, 80]. A Finnish study revealed that only half of unemployed were able to work without any support [28]. This, in line with our results, suggests that a share of unemployed job seekers might be unable to work and could instead be referred to disability pension or rehabilitation services. In Finland, unemployed people may receive unemployment benefits to support their livelihood, but these benefits are only paid to those who are capable of working and willing to accept job offers. If their work ability is reduced, unemployed people should apply for sickness allowance, rehabilitation services, or disability pensions, all of which require a medical certificate.

Unemployed persons’ service needs are assessed in employment services. This requires not only the ability to identify individual needs but also comprehensive knowledge of the service system [80]. Rather than focusing on the assessment of work ability, the expertise of employment officers tends to focus on job placement. However, if health-related issues are not acknowledged, unemployed persons may lack the motivation or capacity to engage with the services offered [81]. Unemployed persons may recognize their work ability as poor but still do not perceive a need for healthcare services; consequently, there is a need to strengthen the connection between work ability and healthcare. Employment service officials should communicate clearly to unemployed persons that healthcare services are available to support their work ability. At the same time, the healthcare system must have the expertise to provide such support [82].

Effective and timely use of healthcare services is important, as delays in treatment can lead to undiagnosed or untreated illnesses. Underuse also complicates the recognition of work disability [83]. In addition to being less likely than employed people to receive a sickness allowance, unemployed people receive less support for their work ability during the sickness allowance period [84]. As a result, their need for rehabilitation often remains unrecognized [79]. Untreated health issues may also lead to higher long-term costs, such as increased emergency care use [85], hospitalisations, or work disability. Being excluded from healthcare services may contribute to increased social isolation and a decline in overall quality of life [86, 87]. Consequently, ensuring timely access to services supports both individual well-being and societal sustainability. However, structural and economic factors may further hinder access to healthcare. Austerity measures have increased unmet healthcare needs, particularly among vulnerable groups such as unemployed people [88]. These unmet needs not only are a barrier to care but also may foster more critical attitudes toward the healthcare system itself [89], potentially reinforcing disengagement from and underuse of services.

### Strengths and limitations

This study has several distinct strengths and limitations. First, it utilized a nationally representative data of the Finnish adult population. The indicators used were validated, well-established, and widely used in population studies. In addition, inverse population weights were used to reduce bias by adjusting for differences in selection probability and nonparticipation [58]. Consequently, the data used included a high number of participants, which enabled the examination of potential confounders using a wide range of background and health-related variables. The limitations of the study are related mainly to the nature of survey questionnaires. Most measures were self-reported, which increases the likelihood of measurement bias, for example by recall bias [90]. If those with the highest or more complex care needs under-reported their care needs or did not respond to the survey at all, the estimates would be biased downwards. Another limitation is the potential mismatch in timing between the outcome and explanatory variables, as the outcomes of interest refer to the past twelve months, whereas the health-related factors were collected at the time of response. Furthermore, the question on no need for or unmet needs reflected only the situation during the 12 months preceding the questionnaire, and we had no information on how the need for healthcare services develops over time, including long-term service use and health consequences [47]. Finally, the study design was cross-sectional; therefore no causal interpretations can be made.

### Policy implications

To address the health disparities faced by unemployed people, it is essential to promote their health literacy and awareness of personal health issues. The findings emphasize the need for targeted healthcare interventions for unemployed people to prevent undiagnosed or untreated conditions and to ensure better utilization of available support systems, such as sickness benefits and rehabilitation services. Healthcare services should be designed to meet the specific needs of unemployed people, not only to improve individual well-being but also to reduce long-term healthcare costs and support re-employment. Efforts should focus on improving the work ability of unemployed individuals, for example through integrated services, multidisciplinary teamwork, and rehabilitation. Additionally, healthcare professionals should be provided with more knowledge and tools to assess and support the work ability of unemployed clients.

## Conclusion

There were minor differences between unemployed and employed people in terms of not needing healthcare services. However, when accounting for their poorer health, unemployed people were much more likely to report no need. Among those who did need healthcare services, unemployed people more often experienced unmet needs, although this was at least partly explained by their poorer health-related factors, especially poor work ability and long-term illness. Several health-related factors were associated with unmet needs for healthcare services in general, indicating that the Finnish primary service system has difficulties offering healthcare services according to need, as the unmet needs are influenced by health problems and social determinants of health, such as unemployment.

Future studies should explore the consequences of unmet healthcare need among unemployed people, particularly how unmet need affect their health and re-employment. There is also a need to identify which specific services or care pathways most frequently contribute to unmet healthcare needs among unemployed people. It is unclear whether gaps exist in access to general practitioners, or whether the unmet need is more pronounced in multidisciplinary services or rehabilitation.

## Data Availability

The data that support the findings of this study are available from the centralized data permit authority Findata (email: [info@findata.fi], tel: +358295246500) but restrictions apply to the availability of these data, which were used under licence for the current study, and are not publicly available. Data are however available from the authors upon reasonable request and with the permission of Findata.

## References

[1] Rechel B, Grundy E, Robine J-M, Cylus J, Mackenbach JP, Knai C, et al. Ageing in the European union. Lancet. 2013;381:1312–22. 10.1016/S0140-6736(12)62087-X. 23541057 10.1016/S0140-6736(12)62087-X

[2] Jóźwiak J, Kotowska IE. Decreasing birth rates in europe: reasons and remedies. Eur View. 2008;7:225–36. 10.1007/s12290-008-0062-6.

[3] Muszyńska MM, Rau R. The Old-Age healthy dependency ratio in Europe. Popul Ageing. 2012;5:151–62. 10.1007/s12062-012-9068-6. 10.1007/s12062-012-9068-6 PMC3412045 22924086

[4] Bloom DE, Chatterji S, Kowal P, Lloyd-Sherlock P, McKee M, Rechel B, et al. Macroeconomic implications of population ageing and selected policy responses. Lancet. 2015;385:649–57. 10.1016/S0140-6736(14)61464-1. 25468167 10.1016/S0140-6736(14)61464-1 PMC4469267

[5] Karanikolos M, Mladovsky P, Cylus J, Thomson S, Basu S, Stuckler D, et al. Financial crisis, austerity, and health in Europe. Lancet. 2013;381:1323–31. 10.1016/S0140-6736(13)60102-6. 23541059 10.1016/S0140-6736(13)60102-6

[6] Stuckler D, Reeves A, Loopstra R, Karanikolos M, McKee M. Austerity and health: the impact in the UK and Europe. Eur J Pub Health. 2017;27 suppl4:18–21. 10.1093/eurpub/ckx167. 29028245 10.1093/eurpub/ckx167 PMC5881725

[7] Strong K, Mathers C, Leeder S, Beaglehole R. Preventing chronic diseases: how many lives can we save? Lancet. 2005;366:1578–82. 10.1016/S0140-6736(05)67341-2. 16257345 10.1016/S0140-6736(05)67341-2

[8] Reeves A, McKee M, Stuckler D. The attack on universal health coverage in europe: recession, austerity and unmet needs. Eur J Pub Health. 2015;25:364–5. 10.1093/eurpub/ckv040. 25999461 10.1093/eurpub/ckv040 PMC4440451

[9] UN. World Social Report 2023: Leaving no one behind in an ageing world. 2023.

[10] McKee-Ryan F, Song Z, Wanberg CR, Kinicki AJ. Psychological and physical Well-Being during unemployment: A Meta-Analytic study. J Appl Psychol. 2005;90:53–76. 10.1037/0021-9010.90.1.53. 15641890 10.1037/0021-9010.90.1.53

[11] Böckerman P, Ilmakunnas P. Unemployment and self-assessed health: evidence from panel data. Health Econ. 2009;18:161–79. 10.1002/hec.1361. 18536002 10.1002/hec.1361

[12] Paul KI, Moser K. Unemployment impairs mental health: Meta-Analyses. J Vocat Behav. 2009;74:264–82. 10.1016/j.jvb.2009.01.001.

[13] Schmitz H. Why are the unemployed in worse health? The causal effect of unemployment on health. Labour Econ. 2011;18:71–8.

[14] Silver SR, Li J, Quay B. Employment status, unemployment duration, and health-related metrics among US adults of prime working age: behavioral risk factor surveillance System, 2018–2019. Am J Ind Med. 2022;65:59–71. 10.1002/ajim.23308. 34748231 10.1002/ajim.23308 PMC8678322

[15] Junna L, Moustgaard H, Martikainen P. Health-related selection into employment among the unemployed. BMC Public Health. 2022;22:657. 10.1186/s12889-022-13023-0. 35382786 10.1186/s12889-022-13023-0 PMC8985275

[16] Picchio M, Ubaldi M. Unemployment and health: A meta-analysis. J Economic Surveys. 2024;38:1437–72. 10.1111/joes.12588.

[17] Pharr JR, Moonie S, Bungum TJ. The impact of unemployment on mental and physical health, access to health care and health risk behaviors. Int Sch Res Notices. 2011;2012:e483432. 10.5402/2012/483432.

[18] Leemann L, Nousiainen M, Keto-Tokoi A, Isola A-M. Osallisuuden Kokemus aikuisväestössä. In: Karvonen S, Kestilä L, Saikkonen P, editors. Suomalaisten hyvinvointi 2022. Helsinki: Terveyden ja hyvinvoinnin laitos; 2022. pp. 94–113.

[19] Ilmarinen J, Gould R, Järvikoski A, Järvisalo J. Diversity of work ability. In: Gould R, Ilmarinen J, Järvisalo J, Koskinen S, editors. Dimensions of work ability. Results of health 2000 survey. Helsinki: Finnish Centre for Pensions, The Social Insurance Institution, National Public Health Institute, Finish Institute of Occupational Health; 2008. pp. 13–24.

[20] Roelfs DJ, Shor E, Davidson KW, Schwartz JE. Losing life and livelihood: a systematic review and meta-analysis of unemployment and all-cause mortality. Soc Sci Med. 2011;72:840–54. 10.1016/j.socscimed.2011.01.005. 21330027 10.1016/j.socscimed.2011.01.005 PMC3070776

[21] Levesque J-F, Harris MF, Russell G. Patient-centred access to health care: conceptualising access at the interface of health systems and populations. Int J Equity Health. 2013;12:18. 10.1186/1475-9276-12-18. 23496984 10.1186/1475-9276-12-18 PMC3610159

[22] Roberts T, Miguel Esponda G, Krupchanka D, Shidhaye R, Patel V, Rathod S. Factors associated with health service utilisation for common mental disorders: a systematic review. BMC Psychiatry. 2018;18:262. 10.1186/s12888-018-1837-1. 30134869 10.1186/s12888-018-1837-1 PMC6104009

[23] Li K, Lorgelly P, Jasim S, Morris T, Gomes M. Does a working day keep the Doctor away? A critical review of the impact of unemployment and job insecurity on health and social care utilisation. Eur J Health Econ. 2023;24:179–86. 10.1007/s10198-022-01468-4. 35522390 10.1007/s10198-022-01468-4 PMC9985560

[24] Hoon E, Pham C, Beilby J, Karnon J. Unconnected and out-of-sight: identifying health care non-users with unmet needs. BMC Health Serv Res. 2017;17:80. 10.1186/s12913-017-2019-4. 28122546 10.1186/s12913-017-2019-4 PMC5264445

[25] Rosato M, Tseliou F, O’Reilly D. Unmet need for chronic mental ill health: A population-based record linkage study. Int J Popul Data Sci. 2019;4:1122. 10.23889/ijpds.v4i1.1122. 34095538 10.23889/ijpds.v4i1.1122 PMC8142951

[26] Nurmela KS, Heikkinen VH, Ylinen AM, Uitti JA, Virtanen PJ. Healthcare attendance styles among long-term unemployed people with substance-related and mood disorders. Public Health. 2020;186:211–6. 10.1016/j.puhe.2020.07.030. 32861086 10.1016/j.puhe.2020.07.030

[27] Åhs A, Burell G, Westerling R. Care or not care–that is the question: predictors of healthcare utilisation in relation to employment status. Int J Behav Med. 2012;19:29–38. 10.1007/s12529-010-9129-2. 21128042 10.1007/s12529-010-9129-2

[28] Kerätär R, Taanila A, Jokelainen J, Soukainen J, Ala-Mursula L. Work disabilities and unmet needs for health care and rehabilitation among jobseekers: a community-level investigation using multidimensional work ability assessments. Scand J Prim Health Care. 2016;34:343–51. 10.1080/02813432.2016.1248632. 27804309 10.1080/02813432.2016.1248632 PMC5217281

[29] Madureira-Lima J, Reeves A, Clair A, Stuckler D. The great recession and inequalities in access to health care: a study of unemployment and unmet medical need in Europe in the economic crisis. Int J Epidemiol. 2018;47:58–68. 10.1093/ije/dyx193. 29024999 10.1093/ije/dyx193 PMC5837221

[30] Fjær EL, Stornes P, Borisova LV, McNamara CL, Eikemo TA. Subjective perceptions of unmet need for health care in Europe among social groups: Findings from the European social survey (2014) special module on the social determinants of health. European Journal of Public Health. 2017;27 suppl_1:82–9. 10.1093/eurpub/ckw219 10.1093/eurpub/ckw219 28355635

[31] Lindstrӧm C, Rosvall M, Lindstrӧm M. Socioeconomic status, social capital and self-reported unmet health care needs: A population-based study. Scand J Public Health. 2017;45:212–21. 10.1177/1403494816689345. 28443488 10.1177/1403494816689345

[32] Moran V, Suhrcke M, Ruiz-Castell M, Barré J, Huiart L. Investigating unmet need for healthcare using the European health interview survey: a cross-sectional survey study of Luxembourg. BMJ Open. 2021;11:e048860. 10.1136/bmjopen-2021-048860. 34344682 10.1136/bmjopen-2021-048860 PMC8336210

[33] Tynkkynen L-K, Keskimäki I, Karanikolos M, Finland. Health system summary 2024. European Observatory on Health Systems and Policies; 2024.

[34] Holster T, Nguyen L, Häkkinen U. The role of occupational health care in ambulatory health care in Finland. Nordic J Health Econ. 2022;10:61–81. 10.5617/njhe.8561.

[35] Virtanen P, Kivimäki M, Vahtera J, Koskenvuo M. Employment status and differences in the one-year coverage of physician visits: different needs or unequal access to services? BMC Health Serv Res. 2006;6:123. 10.1186/1472-6963-6-123. 17014702 10.1186/1472-6963-6-123 PMC1618837

[36] Klavus J, Forma L, Partanen J, Rissanen P. Need and utilization of primary health care among long-term unemployed Finns. J Health Social Sci. 2018;3:231–42.

[37] Blomgren J, Virta LJ. Socioeconomic differences in use of public, occupational and private health care: A register-linkage study of a working-age population in Finland. PLoS ONE. 2020;15:e0231792. 10.1371/journal.pone.0231792. 32298356 10.1371/journal.pone.0231792 PMC7162494

[38] Blomgren J, Jäppinen S, Lahdensuo K. Avosairaanhoidon Palvelujen käyttö on Vahvasti Eriytynyt työmarkkina-aseman Mukaan. Lääkärilehti. 2022;77:e30509.

[39] Rinne H, Blomgren J. Use of outpatient healthcare services before and after the onset of unemployment: A register-based propensity score matched study from Finland. PLoS ONE. 2023;18:e0288423. 10.1371/journal.pone.0288423. 37556479 10.1371/journal.pone.0288423 PMC10411812

[40] Lappalainen K, Mattila-Holappa P, Yli-Kaitala K, Hult M, Räsänen K. Pisimpään työttömänä Olleet käyttävät vähiten Terveyskeskuksen Palveluja. Lääkärilehti. 2018;73:2421–8.

[41] Väisänen V, Sinervo L. Työttömien sosiaali- Ja Terveyspalveluiden käyttö Rekisteritietojen Valossa. Helsinki: THL; 2021.

[42] Hakamäki P, Saaristo V, Lindfors P, Ståhl T. Tukityöllistäminen interventiona Ja Sen Vaikutus Perusterveydenhuollon Palvelujen käyttöön. Sosiaalilääketieteellinen Aikakauslehti. 2022;59:351–64. 10.23990/sa.103415.

[43] Saikku P, Väisänen V, Sinervo L, Karvonen S. Työttömien sosiaali- Ja Terveyspalvelujen käyttö Ja Kustannukset. In: Kestilä L, Saikkonen P, editors. Suomalaisten hyvinvointi 2022. Helsinki: Terveyden ja hyvinvoinnin laitos; 2022. pp. 208–25.

[44] Perhoniemi R, Blomgren J. Frequent attenders of three outpatient health care schemes in finland: characteristics and association with long-term sickness absences, 2016–2018. BMC Public Health. 2021;21:870. 10.1186/s12889-021-10866-x. 33957897 10.1186/s12889-021-10866-x PMC8101095

[45] Faces of joblessness in Finland. OECD. 2021. https://www.oecd.org/en/publications/faces-of-joblessness-in-finland_ca3bc4a4-en.html. Accessed 27 Oct 2025.

[46] Allin S, Masseria C. Unmet need as an indicator of health care access. Eurohealth. 2009;15:7–10.

[47] Smith S, Connolly S. Re-thinking unmet need for health care: introducing a dynamic perspective. Health Econ Policy Law. 2020;15:440–57. 10.1017/S1744133119000161. 31018874 10.1017/S1744133119000161

[48] Allin S, Grignon M, Le Grand J. Subjective unmet need and utilization of health care services in canada: what are the equity implications? Soc Sci Med. 2010;70:465–72. 10.1016/j.socscimed.2009.10.027. 19914759 10.1016/j.socscimed.2009.10.027

[49] Rosenberg M, Kowal P, Rahman MM, Okamoto S, Barber SL, Tangcharoensathien V. Better data on unmet healthcare need can strengthen global monitoring of universal health coverage. BMJ. 2023;382:e075476. 10.1136/bmj-2023-075476. 37669794 10.1136/bmj-2023-075476 PMC10477915

[50] Manderbacka K, Muuri A, Keskimäki I, Kaikkonen R, Elovainio M. Mitä tyydyttämätön palvelutarve Kertoo Terveyspalvelujen saatavuudesta? Sosiaalilääketieteellinen Aikakauslehti. 2012;49:4–12.

[51] Ilmarinen KM, Aalto A-M, Muuri AL. Unmet need for and barriers to receiving health care and social welfare services in Finland. Scand J Public Health. 2025;53(Suppl 1):52–63. 10.1177/14034948241299019. 39663697 10.1177/14034948241299019 PMC11951374

[52] Nguyen L, Häkkinen U. Determinants and associated costs of unmet healthcare need and their association with resource allocation. Insights from Finland. Health Policy. 2025;154:105272. 10.1016/j.healthpol.2025.105272. 40015202 10.1016/j.healthpol.2025.105272

[53] Levesque J-F, Pineault R, Hamel M, Roberge D, Kapetanakis C, Simard B, et al. Emerging organisational models of primary healthcare and unmet needs for care: insights from a population-based survey in Quebec Province. BMC Fam Pract. 2012;13:66. 10.1186/1471-2296-13-66. 22748060 10.1186/1471-2296-13-66 PMC3431245

[54] Huang J, Birkenmaier J, Kim Y. Job loss and unmet health care needs in the economic recession: different associations by family income. Am J Public Health. 2014;104:e178–83. 10.2105/AJPH.2014.301998. 25211745 10.2105/AJPH.2014.301998 PMC4202960

[55] Drapeau A, Fleury MJ, Gentil L. Sociodemographic variation in increasing needs for mental health services among Canadian adults from 2002 to 2012. Psychiatr Q. 2019;90:137–50. 10.1007/s11126-018-9607-2. 30338421 10.1007/s11126-018-9607-2

[56] Blázquez-Fernández C, Cantarero-Prieto D, Pérez P. Unmet health care needs among the working-age population: evidence from the great recession in Spain (2008–2012). Sist Sanit Navar. 2024;47:e1093. 10.23938/ASSN.1093. 10.23938/ASSN.1093 PMC11783131 39717952

[57] Alemu FW, Yuan J, Kadish S, Son S, Khan SS, Nulla SM, et al. Social determinants of unmet need for primary care: a systematic review. Syst Rev. 2024;13:252. 10.1186/s13643-024-02647-5. 39358748 10.1186/s13643-024-02647-5 PMC11448019

[58] Sääksjärvi K, Elonheimo HM, Ikonen J, Lehtoranta L, Parikka S, Härkänen T, et al. Cohort profile: healthy Finland survey. Int J Epidemiol. 2025;54:dyae166. 10.1093/ije/dyae166. 10.1093/ije/dyae166 PMC11665929 39715488

[59] The adult population’s well-being and health – Healthy Finland survey. THL. 2025. https://thl.fi/en/statistics-and-data/data-and-services/quality-and-statistical-principles/quality-descriptions/the-adult-population-s-well-being-and-health-healthy-finland-survey. Accessed 19 June 2025.

[60] Questionnaires THL. 2025. https://thl.fi/en/research-and-development/research-and-projects/healthy-finland-survey/information-for-researchers/questionnaires. Accessed 19 June 2025.

[61] Statistics Finland. More employed and unemployed in June 2023 than one year earlier. 2023. https://stat.fi/en/publication/cl89wrk0dsz7i0bw0isvy1fjo. Accessed 27 Oct 2025.

[62] Robine J-M, Jagger C, The Euro-REVES Group. Creating a coherent set of indicators to monitor health across europe: the Euro-REVES 2 project. Eur J Pub Health. 2003;13(Suppl 1):6–14. 10.1093/eurpub/13.suppl_1.6. 14533742 10.1093/eurpub/13.suppl_1.6

[63] Van Oyen H, Bogaert P, Yokota RTC, Berger N. Measuring disability: a systematic review of the validity and reliability of the global activity limitations indicator (GALI). Arch Public Health. 2018;76:25. 10.1186/s13690-018-0270-8. 29881544 10.1186/s13690-018-0270-8 PMC5985596

[64] Ware JEJ, Sherbourne CD. The MOS 36-ltem Short-Form health survey (SF-36): I. Conceptual framework and item selection. Med Care. 1992;30:473. 1593914

[65] The Washington Group. Short Set of Functioning (WG-SS). 2020.

[66] Leemann L, Martelin,Tuija K, Seppo, Härkänen, Tommi, and, Isola A-M. Development and Psychometric Evaluation of the Experiences of Social Inclusion Scale. Journal of Human Development and Capabilities. 2022;23:400–24. 10.1080/19452829.2021.1985440

[67] Elovainio M, Laaksonen M, Sakari K, Aalto A-M, Jääskeläinen T, Rissanen H, et al. Association of short poor work ability measure with increased mortality risk: a prospective multicohort study. BMJ Open. 2022;12:e065672. 10.1136/bmjopen-2022-065672. 36549734 10.1136/bmjopen-2022-065672 PMC9791446

[68] Saunders JB, Aasland OG, Babor TF, de la Fuente JR, Grant M. Development of the alcohol use disorders identification test (AUDIT): WHO collaborative project on early detection of persons with harmful alcohol Consumption–II. Addiction. 1993;88:791–804. 10.1111/j.1360-0443.1993.tb02093.x. 8329970 10.1111/j.1360-0443.1993.tb02093.x

[69] Härkänen T, Kaikkonen R, Virtala E, Koskinen S. Inverse probability weighting and doubly robust methods in correcting the effects of non-response in the reimbursed medication and self-reported turnout estimates in the ATH survey. BMC Public Health. 2014;14:1150. 10.1186/1471-2458-14-1150. 25373328 10.1186/1471-2458-14-1150 PMC4246429

[70] Corscadden L, Levesque JF, Lewis V, Strumpf E, Breton M, Russell G. Factors associated with multiple barriers to access to primary care: an international analysis. Int J Equity Health. 2018;17:28. 10.1186/s12939-018-0740-1. 29458379 10.1186/s12939-018-0740-1 PMC5819269

[71] Pinto AD, Hassen N, Craig-Neil A. Employment interventions in health settings: A systematic review and synthesis. Annals Family Med. 2018;16:447–60. 10.1370/afm.2286. 10.1370/afm.2286 PMC6130994 30201643

[72] Staiger T, Waldmann T, Krumm S, Rüsch N. Stigma and poor mental health literacy as barriers to service use among unemployed people with mental Illness – A qualitative study. Eur Psychiatry. 2016;33:S487–487. 10.1016/j.eurpsy.2016.01.1786.

[73] Huhtakangas M, Tuomikoski A-M, Kyngäs H, Kanste O. Frequent attenders’ experiences of encounters with healthcare personnel: A systematic review of qualitative studies. Nurs Health Sci. 2021;23:53–68. 10.1111/nhs.12784. 33034401 10.1111/nhs.12784

[74] Tiirinki H, Sulander J, Sinervo T, Halme S, Keskimäki I. Integrating health and social services in finland: regional approaches and governance models. Int J Integr Care. 2022;22:18. 10.5334/ijic.5982. 36186511 10.5334/ijic.5982 PMC9479674

[75] Deri C. Social networks and health service utilization. J Health Econ. 2005;24:1076–107. 10.1016/j.jhealeco.2005.03.008. 16139910 10.1016/j.jhealeco.2005.03.008

[76] Åhs AMH, Westerling R. Health care utilization among persons who are unemployed or outside the labour force. Health Policy. 2006;78:178–93. 10.1016/j.healthpol.2005.10.010. 16343685 10.1016/j.healthpol.2005.10.010

[77] Coomer K, Houdmont J. Occupational health professionals’ knowledge, Understanding and use of work ability. Occup Med. 2013;63:405–9. 10.1093/occmed/kqt070. 10.1093/occmed/kqt070 PMC3748362 23771875

[78] Hinkka K, Niemelä M, Autti-Rämö I, Palomäki H. Physicians’ experiences with sickness absence certification in Finland. Scand J Public Health. 2019;47:859–66. 10.1177/1403494818758817. 29485317 10.1177/1403494818758817

[79] Oivo T, Kerätär R. Osatyökykyisten reitit työllisyyteen - etuudet, palvelut, Tukitoimet: Selvityshenkilöiden Raportti. Helsinki: Sosiaali- ja terveysministeriö; 2018.

[80] Karjalainen P, Liukko E. Työ- Ja Toimintakyvyn edistäminen. In: Koivisto J, Tiirinki H, editors. (Toim.) Monialaisen palvelutarpeen Tunnistaminen sosiaali-, tarveys- Ja työvoimapalveluissa. Helsinki: Valtioneuvoston kanslia; 2020. pp. 60–85.

[81] Blomgren S, Saikku P. Heikossa työmarkkina-asemassa Oleville Suunnattujen Palvelujen Yhteensovittaminen Ja kehittämistarpeet – asiakkaiden Ja Johdon näkökulma. In: Juvonen-Posti P, Saikku P, Turunen J, editors. Elinikäistä Osallistumista Vai elämää työ edellä? Työikäisten monialaisten palveluiden Yhteensovittaminen Ja Vaikuttavuuden arviointi -loppuraportti. Helsinki: Valtioneuvoston kanslia; 2020. pp. 57–89.

[82] Hult M, Pietilä A-M, Saaranen T. Improving employment opportunities of the unemployed by health and work ability promotion in Finland. Health Promot Int. 2020;35:518–26. 10.1093/heapro/daz048. 31132120 10.1093/heapro/daz048

[83] Kuntoutuksen uudistamiskomitea. Kuntoutuksen Uudistamiskomitean Ehdotukset kuntoutusjärjestelmän uudistamiseksi. Helsinki: Sosiaali- ja terveysministeriö; 2017.

[84] Blomgren J, Laaksonen M, Perhoniemi R, Rinne H. Sairauspäivärahakausien tarkistuspisteet: Kuntoutuksen Ja työhön Paluun Toteutuminen. Helsinki: Valtioneuvoston kanslia; 2023.

[85] Bieler G, Paroz S, Faouzi M, Trueb L, Vaucher P, Althaus F, et al. Social and medical vulnerability factors of emergency department frequent users in a universal health insurance system. Acad Emerg Med. 2012;19:63–8. 10.1111/j.1553-2712.2011.01246.x. 22221292 10.1111/j.1553-2712.2011.01246.x

[86] Ju YJ, Kim TH, Han K-T, Lee HJ, Kim W, Ah Lee S, et al. Association between unmet healthcare needs and health-related quality of life: a longitudinal study. Eur J Pub Health. 2017;27:631–7. 10.1093/eurpub/ckw264. 28122811 10.1093/eurpub/ckw264

[87] Yang JM, Lee SK, Kim JH. Association between objective social isolation and unmet medical needs: A nationwide Cross-sectional study in Korea. JPMPH. 2024;57:242–51. 10.3961/jpmph.23.516. 10.3961/jpmph.23.516 PMC11164600 38697912

[88] Doetsch JN, Schlösser C, Barros H, Shaw D, Krafft T, Pilot E. A scoping review on the impact of austerity on healthcare access in the European union: rethinking austerity for the most vulnerable. Int J Equity Health. 2023;22:3. 10.1186/s12939-022-01806-1. 36604705 10.1186/s12939-022-01806-1 PMC9815671

[89] Aalto A-M, Muuri A, Ilmarinen K, Ikonen J. Sosiaali- Ja terveyspalvelujärjestelmä toimintaympäristön myllerryksessä - miten on käynyt väestön kokemalle luottamukselle? In: Karvonen S, Kestilä L, editors. Suomalaisten hyvinvointi 2022. Helsinki: Terveyden ja hyvinvoinnin laitos; 2022. pp. 289–309.

[90] Coughlin SS. Recall bias in epidemiologic studies. J Clin Epidemiol. 1990;43:87–91. 10.1016/0895-4356(90)90060-3. 2319285 10.1016/0895-4356(90)90060-3

